# Obese Children Do Not Need to Increase Their Physical Activity Any More than Their Lean Counterparts Do

**DOI:** 10.3389/fped.2016.00035

**Published:** 2016-04-13

**Authors:** Greg Peter Traversy, Jean-Philippe Chaput

**Affiliations:** ^1^Healthy Active Living and Obesity Research Group, Children’s Hospital of Eastern Ontario Research Institute, Children’s Hospital of Eastern Ontario, Ottawa, ON, Canada; ^2^Faculty of Health Sciences, School of Human Kinetics, University of Ottawa, Ottawa, ON, Canada; ^3^Department of Pediatrics, Faculty of Medicine, University of Ottawa, Ottawa, ON, Canada

**Keywords:** obese, obesity, children, pediatric population, physical activity, energy expenditure

## Introduction

The relationship between child physical activity (PA) levels and obesity has been studied extensively (1, 2). Studies have often shown that obese children and adolescents spend more time in sedentary behavior and less time in non-sedentary behavior, such as moderate-to-vigorous physical activity (MVPA) (3, 4). Accordingly, at least one review of cross-sectional studies examining the link between PA and obesity has found higher levels of PA associated with lower measures of adiposity (2). Some individuals may interpret these suggestions as meaning that obese children need to increase their PA levels to attain a “healthy” weight. Despite the fact that PA generally produces very modest weight loss (randomized controlled trials typically show less than 2 kg of weight loss from exercise interventions), the perception exists, among laypeople and health professionals alike, that obese individuals are lazy and need to “get off the couch” and that a lack of exercise is an important cause of child obesity (5–7).

Is a lack of activity really driving the difference in body composition between normal weight and obese children? It has been suggested that although obese children may move less than normal weight children, they are likely expending just as much, if not more, energy from PA due to a higher metabolic cost of movement (they have a larger body to carry) (5). Additionally, PA levels are extremely low among normal weight and obese children alike, whereas PA remains important for overall physical and mental health (8). This suggests that PA needs to be promoted in all children regardless of weight status, and the view that obese children need to be more targeted for extra PA because of their weight is misleading. We intend to explore this suggestion further and suggest that although patterns of PA may differ between overweight/obese and normal weight children, these are not sufficient to explain differences in weight status. Alternative interpretations of the relationship between obesity and PA are also discussed.

## Physical Activity and Obesity

Studies that examined free-living behavior found greater sedentary behavior and less MVPA and sports activity in obese children compared to lean children (3, 4, 9). A review of cross-sectional studies reported that 79% of the studies examined found negative correlations between PA levels and adiposity (2). A review of prospective studies also found that PA levels are associated with lower body fat accumulation and that MVPA is associated with lower BMI at follow-up in some studies, but not others (10). This review suggested that genetic predisposition and low PA levels are factors closely associated with the development of fatness (10). However, other reviews have suggested that the evidence for a relationship between PA and adiposity is less unanimous (1). Wilks et al. (1) found that the majority of prospective studies they examined failed to find a negative association between PA and changes in adiposity. One study even found a positive association, such that greater total baseline energy expenditure (EE) was associated with increase in adiposity (1). Therefore, it is not clear whether one would always expect to find that low PA predicts greater adiposity, once important confounders (such as genetic, social, physical, and mental factors) are taken into consideration.

Large national health studies can be used to provide a good representative estimate of PA in obese and lean children. The results of the Canadian Health Measures Survey (CHMS), for example, show that obese boys achieve significantly lower average daily step counts compared to non-obese boys (10,256 vs. 12,584) (9). Conversely, in this same survey, obese girls were found to obtain more steps per day than non-obese ones (11,159 vs. 10,224) (9). However, step counts do not provide insight into activity intensity. CHMS accelerometer data showed that obese and overweight children did not engage in significantly different amounts of objectively measured sedentary behavior compared to lean children. However, overweight/obese boys (but not girls) engaged in significantly less MVPA per day compared to lean children (9). Data from the National Health and Nutrition Examination Survey (NHANES) in the USA suggest that normal weight youth aged 6–11, 12–15, and 16–19 years recorded on average of 658, 456, and 390 accelerometer counts per minute, respectively (11). Obese youth recorded 525, 417, and 365 counts per minute, respectively, for the same age groups. The differences between obese and lean children were significant for 6- to 11- and 16- to 19-year olds, but not 12- to 15-year olds (11). The accelerometer data corresponded to 248, 50, and 9 min in sedentary, moderate, and vigorous-intensity activity per day, respectively, for normal weight youth. In obese youth, this corresponded to 254, 39, and 5 min per day in sedentary, moderate, and vigorous-intensity activity, respectively (11). Only the differences in moderate and vigorous-intensity activity levels were statistically significant (11).

Thus, it does appear that overweight/obese children may engage in less MVPA and more light intensity PA than lean children. However, measures of PA, such as pedometer steps or accelerometer counts, act as indices of EE, which ultimately impacts energy balance and thereby weight gain or weight loss. Thus, it is important to examine whether or not differences in PA levels between obese and lean children and adolescents (when they exist) truly correspond to differences in EE, and whether these differences may be preventing obese children from attaining a normal weight.

## Obese and Lean – Who Burns More Calories?

There have been a number of laboratory studies examining EE between obese and lean individuals during aspects of the daily sleep–wake cycle (3, 4). These experiments have shown that overweight/obese individuals expend more energy at rest, during sleep, during sedentary activities, and during miscellaneous activities due to the increased energy costs of moving a larger body (3, 4, 12).

Although obese youth may expend more energy during exercise under laboratory conditions, this may not correspond to free-living conditions. Studies of daily free-living EE have found lower expenditure from MVPA in obese children and adolescents (but not light PA). However, total PA EE has not been found to be significantly different between groups (3, 12). In some studies, estimates of PA EE are even higher in obese children (4).

The results of these experiments, taken with results such as those of the CHMS, suggest that obese individuals, although displaying different patterns of activity, are likely expending just as much, if not more, energy on a daily basis than lean ones. Their lack of MVPA or “laziness,” as some may describe it, does not explain the difference in body composition between them and their lean peers. Also, there may be other explanations for why PA and adiposity sometimes negatively correlate with one another.

## Mitigating Factors Between PA and Adiposity

When considering the link between PA and adiposity, there are a number of important factors to consider. These could include genetic factors, such as polymorphisms influencing reward pathways; social factors, such as parental or peer influence; physical factors, such as the built environment; dietary habits; levels of sedentary behavior; and personal factors, such as fitness, confidence, and motivation (10, 13). Mental health issues are also of importance, as depression or exposure to bullying potentially faced by obese children has the potential to influence some of these factors. Additionally, there is evidence to suggest that the association between PA and obesity is not consistent across different ethnic groups and genders (14). One should also consider overall energy balance (between caloric intake and expenditure) as opposed to considering them independently.

Given that studies finding negative associations between PA and adiposity are largely cross-sectional in nature, the potential for residual confounding and reverse causation should always be considered (2). In other words, rather than concluding that increased PA protects against obesity, one could also conclude that being obese may influence decisions about being physically active. A recent longitudinal study provides evidence for such an association. In 8- to 11-year-old children, fat mass index at baseline predicted lower PA, lower MVPA, and higher sedentary behavior over 200 days of follow-up (15). However, the opposite was not found to be true. None of the movement behaviors assessed at baseline predicted changes in fat mass index (15). Thus, the authors suggested that “adiposity is a better predictor of PA and sedentary behavior changes than the other way around” (15).

## Conclusion

Although lower levels of PA have been found to correlate with obesity in some studies, urging obese children to “get off the couch” to address their excess weight is too simplistic and unlikely to result in meaningful weight losses. Furthermore, such a suggestion may undermine children’s motivation toward healthy active living. Evidence is showing that obese children and adolescents are expending as much, if not more energy, than their lean peers (5). However, what is clear is that we are facing a global epidemic of physical inactivity, i.e., the vast majority of children (lean and obese) do not meet the recommended amount of PA required each day for health benefits (8). PA benefits cardiovascular and other physical health outcomes (even in those classified as obese), cognitive function, and optimal development of social and motor skills, mental health, self-esteem, and quality of life (8, 16). Thus, increased PA should be promoted in children of all sizes, more so to promote overall health, than to bring body weight to a supposedly “ideal” spot.

## Author Contributions

The authors GT and J-PC both contributed to the conception, writing, and editing of the manuscript.

## Conflict of Interest Statement

The authors prepared this article in the absence of any commercial or financial relationships that could be construed as a potential conflict of interest.
